# 4-[4-(3-Methoxy­benzamido)phen­oxy]-*N*-methyl­picolinamide

**DOI:** 10.1107/S1600536809055688

**Published:** 2010-01-30

**Authors:** Na-Na Meng, Ting-Ting Huang, De-Kuan Li, Wen-Xin Zhang, Luo-Ting Yu

**Affiliations:** a State Key Laboratory of Biotherapy and Cancer Center, West China Hospital, West China Medical School, Sichuan University, Chengdu 610041, People’s Republic of China; b Key Laboratory of Bio-resources and Eco-environment of the Ministry of Education, College of Life Science, Sichuan University, Chengdu 610041, People’s Republic of China; c Department of Pharmaceutical and Bioengineering, School of Chemical Engineering, Sichuan University, Chengdu 610065, People’s Republic of China

## Abstract

In the title compound, C_21_H_19_N_3_O_4_, the central benzene ring makes dihedral angles of 78.54 (6) and 75.30 (6)° with the pyridine and 3-methoxy­phenyl rings, respectively. An intra­molecular N—H⋯N interaction occurs, generating an *S*(?). The crystal packing shows inter­molecular N—H⋯O hydrogen-bonding inter­actions between the N—H groups and the O atoms of the 3-methoxy­phenyl ring and the carbonyl groups of the amide functions. Inter­molecular C—H⋯O inter­actions are also present.

## Related literature

For related compounds and their biological activity, see: Khire *et al.* (2004 ▶); Dominguez *et al.* (2007 ▶).
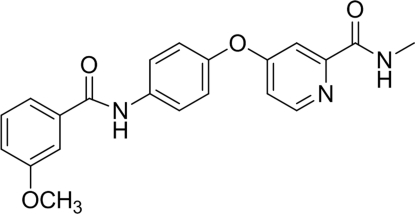


## Experimental

### 

#### Crystal data

C_21_H_19_N_3_O_4_
*M* *_r_* = 377.39
Triclinic, 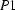

*a* = 5.0915 (10) Å
*b* = 8.3251 (17) Å
*c* = 11.611 (2) Å
α = 71.29 (3)°
β = 87.74 (3)°
γ = 76.10 (3)°
*V* = 452.14 (16) Å^3^
*Z* = 1
Mo *K*α radiation
μ = 0.10 mm^−1^
*T* = 113 K
0.34 × 0.29 × 0.19 mm

#### Data collection

Rigaku Saturn CCD area-detector diffractometer
Absorption correction: multi-scan (*ABSCOR*; Higashi, 1995 ▶) *T*_min_ = 0.968, *T*_max_ = 0.982
3733 measured reflections
2108 independent reflections
1811 reflections with *I* > 2σ(*I*)
*R*_int_ = 0.026

#### Refinement

*R*[*F* ^2^ > 2σ(*F* ^2^)] = 0.035
*wR*(*F* ^2^) = 0.085
*S* = 1.10
2108 reflections
263 parameters
3 restraints
H atoms treated by a mixture of independent and constrained refinement
Δρ_max_ = 0.22 e Å^−3^
Δρ_min_ = −0.24 e Å^−3^

### 

Data collection: *CrystalClear* (Rigaku/MSC, 2005 ▶); cell refinement: *CrystalClear*; data reduction: *CrystalClear*; program(s) used to solve structure: *SHELXS97* (Sheldrick, 2008 ▶); program(s) used to refine structure: *SHELXL97* (Sheldrick, 2008 ▶); molecular graphics: *ORTEPIII* (Burnett & Johnson, 1996 ▶); software used to prepare material for publication: *PLATON* (Spek, 2009 ▶).

## Supplementary Material

Crystal structure: contains datablocks global, I. DOI: 10.1107/S1600536809055688/om2308sup1.cif

Structure factors: contains datablocks I. DOI: 10.1107/S1600536809055688/om2308Isup2.hkl

Additional supplementary materials:  crystallographic information; 3D view; checkCIF report

## Figures and Tables

**Table 1:** Hydrogen-bond geometry (Å, °)

*D*—H⋯*A*	*D*—H	H⋯*A*	*D*⋯*A*	*D*—H⋯*A*
N1—H1*N*⋯O2^i^	0.89 (3)	2.08 (3)	2.918 (2)	155 (3)
N3—H3*N*⋯O1^ii^	0.85 (3)	2.38 (3)	3.148 (3)	151 (2)
N3—H3*N*⋯N2	0.85 (3)	2.33 (3)	2.681 (3)	105 (2)
C7—H7*B*⋯O4^iii^	0.98	2.55	3.475 (3)	158

Symmetry codes: (i) 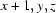; (ii) 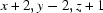; (iii) 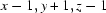.

**Fig. 1.**
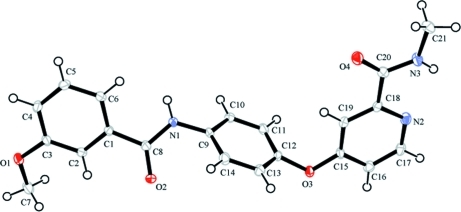
The molecular structure of the title compound, with displacement ellipsoids drawn at the 30% probability level.

**Fig. 2.**
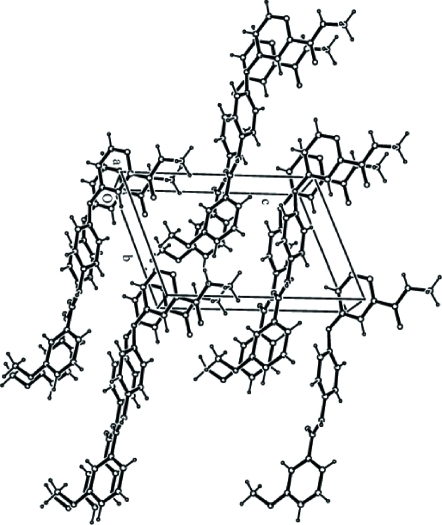
The cell packing of the title compound.

## supplementary crystallographic information

### Comment

Sorafenib is of great importance owing to its antitumor properties (Khire *et al.*, 2004; Dominguez *et al.*, 2007). The title compound, as one of its derivatives, possessed even better *in vitro* anticancer activity against both two tumor cell lines (HCT116 and HEPG2). As a potent antitumor drug, we report here its crystal structure.

In the title molecule, C_21_H_19_N_3_O_4_, (Fig.1), the phenyl ring makes dihedral angles of 78.54 (6)° and 75.30 (6)° with the pyridine ring and the 3-methoxyphenyl ring, respectively. In the crystal structure, intermolecular N—H···O hydrogen-bonding interactions between the N—H and O atoms of 3-methoxyphenyl ring and carbonyl groups of the amide functionalities form an infinite three-dimensional structure (Table 1 and Fig. 2).

### Experimental

To the suspension of anhydrous potassium carbonate (1.635 g,12.5 mmol) and 4-(4-aminophenoxy)-*N*-methylpicolinamide (1.22 g, 5 mmol) in 11.4 ml THF was added dropwise 3-methoxybenzoyl chloride(1.28 g,7.5 mmol). After being stirred at room temperature for 2 h, the mixture was extracted with 90 ml EA and 30 ml water for three times and the combined organic layers were dried over anhydrous Na_2_SO_4_. Then the solution was concentrated under vacuum, and the residue was recrystallized from ethanol to give the title compound. Crystals suitable for X-ray analysis were obtained by slow evaporation from a solution of ethanol.

### Refinement

The two H atoms of N1 and N3 were located in a difference map and refined isotropically. The reminaing H atoms were positioned geometrically (C—H = 0.95–0.98 Å) and refined using a riding model, with *U*_iso_(H) = 1.2–1.5*U*_eq_(C). In the final stages of refinement, Friedel-pair reflections were merged.

### Crystal data

**Table:**

C_21_H_19_N_3_O_4_	*Z* = 1
*M**_r_* = 377.39	*F*(000) = 198
Triclinic, *P*1	*D*_x_ = 1.386 Mg m^−^^3^
*a* = 5.0915 (10) Å	Mo *K*α radiation, λ = 0.71073 Å
*b* = 8.3251 (17) Å	Cell parameters from 1652 reflections
*c* = 11.611 (2) Å	θ = 2.7–27.8°
α = 71.29 (3)°	µ = 0.10 mm^−^^1^
β = 87.74 (3)°	*T* = 113 K
γ = 76.10 (3)°	Block, colourless
*V* = 452.14 (16) Å^3^	0.34 × 0.29 × 0.19 mm

### Data collection

**Table:**

Rigaku Saturn CCD area-detector diffractometer	2108 independent reflections
Radiation source: rotating anode	1811 reflections with *I* > 2σ(*I*)
confocal	*R*_int_ = 0.026
Detector resolution: 7.31 pixels mm^-1^	θ_max_ = 27.8°, θ_min_ = 2.7°
ω and φ scans	*h* = −6→4
Absorption correction: multi-scan (*ABSCOR*; Higashi, 1995)	*k* = −10→10
*T*_min_ = 0.968, *T*_max_ = 0.982	*l* = −14→15
3733 measured reflections	

### Refinement

**Table:**

Refinement on *F*^2^	Primary atom site location: structure-invariant direct methods
Least-squares matrix: full	Secondary atom site location: difference Fourier map
*R*[*F*^2^ > 2σ(*F*^2^)] = 0.035	Hydrogen site location: mixed
*wR*(*F*^2^) = 0.085	H atoms treated by a mixture of independent and constrained refinement
*S* = 1.10	*w* = 1/[σ^2^(*F*_o_^2^) + (0.0481*P*)^2^] where *P* = (*F*_o_^2^ + 2*F*_c_^2^)/3
2108 reflections	(Δ/σ)_max_ < 0.001
263 parameters	Δρ_max_ = 0.22 e Å^−^^3^
3 restraints	Δρ_min_ = −0.24 e Å^−^^3^

### Special details

**Table:**

Geometry. All e.s.d.'s (except the e.s.d. in the dihedral angle between two l.s. planes) are estimated using the full covariance matrix. The cell e.s.d.'s are taken into account individually in the estimation of e.s.d.'s in distances, angles and torsion angles; correlations between e.s.d.'s in cell parameters are only used when they are defined by crystal symmetry. An approximate (isotropic) treatment of cell e.s.d.'s is used for estimating e.s.d.'s involving l.s. planes.
Refinement. Refinement of *F*^2^ against ALL reflections. The weighted *R*-factor *wR* and goodness of fit *S* are based on *F*^2^, conventional *R*-factors *R* are based on *F*, with *F* set to zero for negative *F*^2^. The threshold expression of *F*^2^ > σ(*F*^2^) is used only for calculating *R*-factors(gt) *etc*. and is not relevant to the choice of reflections for refinement. *R*-factors based on *F*^2^ are statistically about twice as large as those based on *F*, and *R*- factors based on ALL data will be even larger.

### Fractional atomic coordinates and isotropic or equivalent isotropic displacement parameters (Å^2^)

**Table:**

	*x*	*y*	*z*	*U*_iso_*/*U*_eq_	
O1	−0.3679 (3)	1.5504 (2)	0.23318 (15)	0.0212 (4)	
O2	−0.1469 (3)	0.9491 (2)	0.56055 (17)	0.0249 (4)	
O3	0.4394 (3)	0.1895 (2)	0.81518 (15)	0.0220 (4)	
O4	1.1345 (3)	0.2263 (2)	1.10678 (16)	0.0230 (4)	
N1	0.3007 (3)	0.9089 (2)	0.60459 (17)	0.0154 (4)	
H1N	0.443 (6)	0.954 (4)	0.579 (3)	0.031 (8)*	
N2	1.0377 (4)	−0.1617 (2)	1.06587 (18)	0.0197 (4)	
N3	1.3326 (4)	−0.0601 (3)	1.20340 (18)	0.0202 (4)	
H3N	1.353 (6)	−0.166 (4)	1.208 (3)	0.025 (7)*	
C1	0.0404 (4)	1.1969 (3)	0.48677 (19)	0.0138 (4)	
C2	−0.1555 (4)	1.2811 (3)	0.3920 (2)	0.0157 (4)	
H2	−0.2683	1.2179	0.3713	0.019*	
C3	−0.1849 (4)	1.4569 (3)	0.3282 (2)	0.0166 (5)	
C4	−0.0230 (4)	1.5503 (3)	0.3610 (2)	0.0186 (5)	
H4	−0.0441	1.6711	0.3181	0.022*	
C5	0.1675 (4)	1.4670 (3)	0.4559 (2)	0.0196 (5)	
H5	0.2754	1.5316	0.4784	0.024*	
C6	0.2036 (4)	1.2891 (3)	0.5190 (2)	0.0167 (5)	
H6	0.3377	1.2318	0.5830	0.020*	
C7	−0.4855 (5)	1.4516 (3)	0.1769 (2)	0.0240 (5)	
H7A	−0.6035	1.3902	0.2340	0.036*	
H7B	−0.3410	1.3663	0.1549	0.036*	
H7C	−0.5919	1.5310	0.1034	0.036*	
C8	0.0553 (4)	1.0091 (3)	0.5533 (2)	0.0163 (4)	
C9	0.3486 (4)	0.7259 (3)	0.66625 (19)	0.0145 (4)	
C10	0.5761 (4)	0.6131 (3)	0.6406 (2)	0.0162 (4)	
H10	0.7046	0.6589	0.5865	0.019*	
C11	0.6152 (4)	0.4336 (3)	0.6942 (2)	0.0190 (5)	
H11	0.7689	0.3560	0.6763	0.023*	
C12	0.4271 (4)	0.3690 (3)	0.7740 (2)	0.0174 (5)	
C13	0.2105 (4)	0.4795 (3)	0.8055 (2)	0.0213 (5)	
H13	0.0892	0.4336	0.8640	0.026*	
C14	0.1710 (4)	0.6586 (3)	0.7512 (2)	0.0204 (5)	
H14	0.0215	0.7356	0.7723	0.025*	
C15	0.6403 (4)	0.0792 (3)	0.8978 (2)	0.0169 (5)	
C16	0.6760 (5)	−0.0977 (3)	0.9182 (2)	0.0194 (5)	
H16	0.5661	−0.1394	0.8757	0.023*	
C17	0.8763 (5)	−0.2126 (3)	1.0024 (2)	0.0210 (5)	
H17	0.9012	−0.3341	1.0161	0.025*	
C18	0.9936 (4)	0.0107 (3)	1.0450 (2)	0.0163 (4)	
C19	0.8000 (4)	0.1369 (3)	0.9625 (2)	0.0161 (4)	
H19	0.7777	0.2578	0.9509	0.019*	
C20	1.1623 (4)	0.0692 (3)	1.1206 (2)	0.0172 (5)	
C21	1.4960 (5)	−0.0250 (3)	1.2878 (2)	0.0220 (5)	
H21A	1.3916	0.0733	1.3122	0.033*	
H21B	1.6598	0.0043	1.2484	0.033*	
H21C	1.5470	−0.1288	1.3599	0.033*	

### Atomic displacement parameters (Å^2^)

**Table:**

	*U*^11^	*U*^22^	*U*^33^	*U*^12^	*U*^13^	*U*^23^
O1	0.0201 (8)	0.0162 (9)	0.0232 (9)	−0.0018 (6)	−0.0079 (6)	−0.0016 (7)
O2	0.0138 (7)	0.0175 (8)	0.0376 (10)	−0.0063 (6)	−0.0052 (7)	0.0017 (7)
O3	0.0228 (8)	0.0134 (8)	0.0260 (9)	−0.0053 (6)	−0.0103 (7)	0.0010 (7)
O4	0.0243 (8)	0.0170 (9)	0.0262 (9)	−0.0024 (6)	−0.0048 (7)	−0.0060 (7)
N1	0.0118 (8)	0.0135 (9)	0.0184 (9)	−0.0030 (7)	0.0003 (7)	−0.0017 (8)
N2	0.0229 (10)	0.0139 (10)	0.0209 (10)	−0.0021 (7)	−0.0008 (8)	−0.0053 (8)
N3	0.0245 (10)	0.0165 (11)	0.0183 (10)	−0.0019 (8)	−0.0045 (8)	−0.0054 (8)
C1	0.0115 (9)	0.0125 (10)	0.0163 (11)	−0.0010 (7)	0.0023 (8)	−0.0047 (8)
C2	0.0125 (9)	0.0155 (11)	0.0195 (11)	−0.0034 (8)	0.0000 (8)	−0.0059 (9)
C3	0.0138 (10)	0.0149 (11)	0.0180 (11)	−0.0004 (8)	−0.0001 (8)	−0.0032 (9)
C4	0.0187 (11)	0.0121 (11)	0.0227 (12)	−0.0029 (8)	0.0017 (9)	−0.0032 (9)
C5	0.0179 (11)	0.0174 (12)	0.0260 (13)	−0.0069 (8)	0.0007 (9)	−0.0085 (10)
C6	0.0136 (10)	0.0171 (12)	0.0189 (11)	−0.0029 (8)	−0.0014 (8)	−0.0056 (9)
C7	0.0237 (12)	0.0198 (12)	0.0272 (12)	0.0009 (9)	−0.0095 (10)	−0.0088 (10)
C8	0.0139 (10)	0.0159 (11)	0.0174 (11)	−0.0024 (8)	0.0000 (8)	−0.0038 (9)
C9	0.0134 (9)	0.0137 (11)	0.0142 (10)	−0.0016 (8)	−0.0038 (8)	−0.0021 (8)
C10	0.0129 (10)	0.0169 (11)	0.0179 (11)	−0.0037 (8)	0.0012 (8)	−0.0043 (9)
C11	0.0168 (10)	0.0166 (11)	0.0217 (11)	−0.0005 (8)	−0.0025 (9)	−0.0057 (9)
C12	0.0200 (11)	0.0130 (11)	0.0164 (11)	−0.0055 (8)	−0.0073 (9)	0.0010 (9)
C13	0.0188 (11)	0.0206 (12)	0.0206 (12)	−0.0061 (8)	0.0018 (9)	−0.0006 (9)
C14	0.0177 (11)	0.0209 (12)	0.0185 (11)	−0.0017 (8)	0.0027 (8)	−0.0031 (9)
C15	0.0168 (10)	0.0147 (11)	0.0146 (11)	−0.0018 (8)	−0.0010 (8)	0.0002 (9)
C16	0.0229 (11)	0.0179 (12)	0.0190 (11)	−0.0069 (9)	0.0001 (8)	−0.0064 (9)
C17	0.0276 (12)	0.0116 (10)	0.0220 (12)	−0.0034 (8)	0.0005 (9)	−0.0040 (9)
C18	0.0182 (11)	0.0152 (11)	0.0143 (10)	−0.0053 (8)	0.0034 (8)	−0.0027 (9)
C19	0.0189 (10)	0.0131 (10)	0.0147 (10)	−0.0043 (8)	0.0014 (8)	−0.0021 (9)
C20	0.0155 (10)	0.0195 (12)	0.0162 (11)	−0.0034 (8)	0.0022 (8)	−0.0060 (9)
C21	0.0206 (11)	0.0253 (13)	0.0203 (11)	−0.0024 (9)	−0.0031 (9)	−0.0091 (10)

### Geometric parameters (Å, °)

**Table:**

O1—C3	1.369 (3)	C7—H7A	0.9800
O1—C7	1.440 (3)	C7—H7B	0.9800
O2—C8	1.237 (3)	C7—H7C	0.9800
O3—C15	1.368 (3)	C9—C14	1.387 (3)
O3—C12	1.402 (3)	C9—C10	1.394 (3)
O4—C20	1.239 (3)	C10—C11	1.389 (3)
N1—C8	1.360 (3)	C10—H10	0.9500
N1—C9	1.424 (3)	C11—C12	1.387 (3)
N1—H1N	0.89 (3)	C11—H11	0.9500
N2—C18	1.340 (3)	C12—C13	1.376 (3)
N2—C17	1.346 (3)	C13—C14	1.387 (3)
N3—C20	1.337 (3)	C13—H13	0.9500
N3—C21	1.450 (3)	C14—H14	0.9500
N3—H3N	0.85 (3)	C15—C16	1.382 (3)
C1—C6	1.391 (3)	C15—C19	1.387 (3)
C1—C2	1.397 (3)	C16—C17	1.386 (3)
C1—C8	1.491 (3)	C16—H16	0.9500
C2—C3	1.386 (3)	C17—H17	0.9500
C2—H2	0.9500	C18—C19	1.387 (3)
C3—C4	1.397 (3)	C18—C20	1.510 (3)
C4—C5	1.380 (3)	C19—H19	0.9500
C4—H4	0.9500	C21—H21A	0.9800
C5—C6	1.396 (3)	C21—H21B	0.9800
C5—H5	0.9500	C21—H21C	0.9800
C6—H6	0.9500		
			
C3—O1—C7	116.81 (17)	C11—C10—C9	120.1 (2)
C15—O3—C12	119.17 (17)	C11—C10—H10	120.0
C8—N1—C9	122.98 (18)	C9—C10—H10	120.0
C8—N1—H1N	116 (2)	C12—C11—C10	119.2 (2)
C9—N1—H1N	118 (2)	C12—C11—H11	120.4
C18—N2—C17	116.25 (18)	C10—C11—H11	120.4
C20—N3—C21	121.4 (2)	C13—C12—C11	121.2 (2)
C20—N3—H3N	122 (2)	C13—C12—O3	118.26 (19)
C21—N3—H3N	117 (2)	C11—C12—O3	120.3 (2)
C6—C1—C2	120.42 (19)	C12—C13—C14	119.4 (2)
C6—C1—C8	122.61 (19)	C12—C13—H13	120.3
C2—C1—C8	116.91 (19)	C14—C13—H13	120.3
C3—C2—C1	119.96 (19)	C13—C14—C9	120.4 (2)
C3—C2—H2	120.0	C13—C14—H14	119.8
C1—C2—H2	120.0	C9—C14—H14	119.8
O1—C3—C2	124.4 (2)	O3—C15—C16	116.9 (2)
O1—C3—C4	115.84 (19)	O3—C15—C19	123.2 (2)
C2—C3—C4	119.77 (19)	C16—C15—C19	119.86 (19)
C5—C4—C3	120.0 (2)	C15—C16—C17	118.2 (2)
C5—C4—H4	120.0	C15—C16—H16	120.9
C3—C4—H4	120.0	C17—C16—H16	120.9
C4—C5—C6	120.8 (2)	N2—C17—C16	123.7 (2)
C4—C5—H5	119.6	N2—C17—H17	118.1
C6—C5—H5	119.6	C16—C17—H17	118.1
C1—C6—C5	119.04 (19)	N2—C18—C19	124.8 (2)
C1—C6—H6	120.5	N2—C18—C20	116.87 (18)
C5—C6—H6	120.5	C19—C18—C20	118.32 (19)
O1—C7—H7A	109.5	C18—C19—C15	117.2 (2)
O1—C7—H7B	109.5	C18—C19—H19	121.4
H7A—C7—H7B	109.5	C15—C19—H19	121.4
O1—C7—H7C	109.5	O4—C20—N3	124.1 (2)
H7A—C7—H7C	109.5	O4—C20—C18	120.93 (19)
H7B—C7—H7C	109.5	N3—C20—C18	115.0 (2)
O2—C8—N1	122.4 (2)	N3—C21—H21A	109.5
O2—C8—C1	120.97 (19)	N3—C21—H21B	109.5
N1—C8—C1	116.65 (19)	H21A—C21—H21B	109.5
C14—C9—C10	119.6 (2)	N3—C21—H21C	109.5
C14—C9—N1	120.77 (18)	H21A—C21—H21C	109.5
C10—C9—N1	119.64 (19)	H21B—C21—H21C	109.5
			
C6—C1—C2—C3	1.0 (3)	C15—O3—C12—C13	−113.2 (2)
C8—C1—C2—C3	178.42 (18)	C15—O3—C12—C11	72.9 (3)
C7—O1—C3—C2	−16.6 (3)	C11—C12—C13—C14	3.7 (3)
C7—O1—C3—C4	164.13 (19)	O3—C12—C13—C14	−170.2 (2)
C1—C2—C3—O1	179.13 (19)	C12—C13—C14—C9	−0.3 (3)
C1—C2—C3—C4	−1.6 (3)	C10—C9—C14—C13	−3.6 (3)
O1—C3—C4—C5	−180.0 (2)	N1—C9—C14—C13	176.2 (2)
C2—C3—C4—C5	0.7 (3)	C12—O3—C15—C16	−168.06 (19)
C3—C4—C5—C6	0.8 (3)	C12—O3—C15—C19	13.8 (3)
C2—C1—C6—C5	0.5 (3)	O3—C15—C16—C17	−179.4 (2)
C8—C1—C6—C5	−176.77 (19)	C19—C15—C16—C17	−1.1 (3)
C4—C5—C6—C1	−1.4 (3)	C18—N2—C17—C16	0.8 (3)
C9—N1—C8—O2	2.7 (3)	C15—C16—C17—N2	0.3 (4)
C9—N1—C8—C1	−177.13 (19)	C17—N2—C18—C19	−1.2 (3)
C6—C1—C8—O2	149.6 (2)	C17—N2—C18—C20	176.7 (2)
C2—C1—C8—O2	−27.7 (3)	N2—C18—C19—C15	0.3 (3)
C6—C1—C8—N1	−30.6 (3)	C20—C18—C19—C15	−177.45 (19)
C2—C1—C8—N1	152.10 (19)	O3—C15—C19—C18	178.95 (19)
C8—N1—C9—C14	−46.8 (3)	C16—C15—C19—C18	0.8 (3)
C8—N1—C9—C10	133.0 (2)	C21—N3—C20—O4	1.9 (3)
C14—C9—C10—C11	4.1 (3)	C21—N3—C20—C18	−176.19 (19)
N1—C9—C10—C11	−175.67 (19)	N2—C18—C20—O4	179.9 (2)
C9—C10—C11—C12	−0.7 (3)	C19—C18—C20—O4	−2.2 (3)
C10—C11—C12—C13	−3.2 (3)	N2—C18—C20—N3	−2.0 (3)
C10—C11—C12—O3	170.56 (18)	C19—C18—C20—N3	175.94 (19)

### Hydrogen-bond geometry (Å, °)

**Table:**

*D*—H···*A*	*D*—H	H···*A*	*D*···*A*	*D*—H···*A*
N1—H1N···O2^i^	0.89 (3)	2.08 (3)	2.918 (2)	155 (3)
N3—H3N···O1^ii^	0.85 (3)	2.38 (3)	3.148 (3)	151 (2)
N3—H3N···N2	0.85 (3)	2.33 (3)	2.681 (3)	105 (2)
C7—H7B···O4^iii^	0.98	2.55	3.475 (3)	158
C14—H14···O2	0.95	2.56	2.887 (3)	100

Symmetry codes: (i) *x*+1, *y*, *z*; (ii) *x*+2, *y*−2, *z*+1; (iii) *x*−1, *y*+1, *z*−1.
